# Selections and Abstracts

**Published:** 1912-11

**Authors:** 


### SELECTIONS AND ABSTRACTS

Dr. C. C. Haskell of the department of experimental medicine of the Lilly laboratories, Indianapolis, recently returned to his office after a summer's work on Staten Island, New York. Dr. Haskell has been connected with the Seaside Hospital of St. John's Guild for several years studying infants' diseases, especially dietary troubles. During the past summer he was house physician and had special opportunity to study the question of infant foods and methods of feeding, as a majority of the patients were infants suffering from gastro-intestinal disturbances.

The American Surgical Association has appointed a committee consisting of Drs. William L. Estes, South Bethlehem, Pa.; Thomas W. Huntington, San Francisco, California; John B. Walker, New York City; Edward Martin, Philadelphia; and John B. Roberts, Chairman, 313 S. 17th Street, Philadelphia to report on the operative and non-operative of closed and open fractures of the long bones and the value of radiography in the study of these injuries. Surgeons, who have published papers relating to this subject within the last ten years, will confer a favor by sending two reprints to the Chairman of the Committee. If no reprints are available, the titles and places of their publication are desired.

John B. Roberts, Chairman
313 S. 17th Street, Philadelphia

### ARMY MEDICAL CORPS EXAMINATIONS.

The Surgeon General of the Army announces that preliminary examinations for the appointment of First Lieutenants in the Army Medical Corps will be held on January 20, 1913, at points to be hereafter designated.

Full information concerning these examinations can be procured upon application to the "Surgeon General, U. S. Army, Washington, D. C." The essential requirements to securing an invitation are that the applicant shall be a citizen of the United States, shall be between 22 and 30 years of age, a graduate of a medical school legally authorized to confer the degree of Doctor of Medicine, shall be of good moral character and habits, and shall have at least one year's hospital training as an interne, after graduation. The examinations will be convened. Due consideration will be given to localities from which applications are received, in order to lessen the traveling expenses of applicants as much as possible.

The examination in subjects of general education (mathematics, geography, history, general literature, and Latin) may be omitted in the cases of applicants holding diplomas from reputable literary or scientific, colleges, normal schools or high schools or graduates of medical schools which require an entrance examination satisfactory to the faculty of the Army Medical School.

In order to protect all necessary arrangements for the examination, applications must be completed and in possession of the Adjutant General at least three weeks before the date of examination. Early attention is therefore enjoined upon all intending applicants. There are at present thirty-five vacancies in the Medical Corps of the Army.

---

### THE FREQUENCY OF DISEASED KIDNEYS

To appreciate the fact that the kidneys are often and seriously affected, one needs to spend some time in the pathological department of any large hospital. A systematic examination of the material derived from the post-mortem table of any such institution will soon disclose the fact that one of the rarest of rare findings is a perfectly healthy kidney. It is true that the examination of the gross material may frequently result in opinions rather favorable, but a systematic microscopic examination will reveal lesions of minute character, and certain definite changes which point to previous disease. Fortunately, nature is prolific in her gifts, and with the kidney substance she has made no exception. It is true that for the ordinary events of life we have somewhat more kidney tissue than is actually needed. There exists, as a wise provision, a surplus of kidney substance which we may call a reserve fund, to be called upon in times of stress and strain. To preserve this surplus, to guard it from damage, to hold it intact for just such emergencies, is one of the duties of the physician.

Nevertheless, in spite of our ever increasing knowledge of the physiology of this important organ, in spite of the warnings from the autopsy table, in spite of the teachings of clinicians that these excretory organs are so frequently diseased during the acute infectious diseases, the medical profession must face the charge that nephritis, Bright's disease, degenerations of the kidney, are almost as great a scourage as the white plague itself.

The medical profession is too often satisfied with a single examination of the urine. Indeed, if we wish to profit by experience, nothing less than a daily examination of the urine during the acute infectious diseases should be considered sufficient. He who relies upon the absence of albumin as sufficient to exclude nephritis commits the same grave mistake as does the practitioner who would exclude pulmonary tuberculosis because of absence of tubercle bacilli in the sputum.

The medical profession recognizes the ethics of the great army of workers in the field for the prevention of tuberculosis. Carefully made autopsies show that diseased kidneys are perhaps the next most frequent of human ailments. In the face of such evidence, and for the great and good reason that the physician alone and unaided cannot stop this frightful cause of death and distress, it becomes the duty of the profession to not only make a confidant of the laity, but more than that, to spread the doctrine of the nature of nephritis, Let the profession teach the doctrine that nephritis is usually, the result of one or more attacks of the infectious diseases. In other words, nephritis should be classed among the preventable diseases. Furthermore, not only should the physician be on the alert to detect evidences of kidney disease during the acute infectious process, but the patient should be kept under observation for a long time after typhoid fever, after acute and subacute tonsillar disease. If any advance has been made in managing tuberculosis, in preventing its spread, it is because physician and laymen are working hand in hand. The public generally will take the same interest with reference to disease of the kidney as it does in disease of the lungs. It needs instruction and needs it badly, and this instruction with reference to nephritis must come from the medical body. It is certain that no spasmodic effort on the part of the physician here and there will accomplish the desired result. The medical profession must not only be a unit in its attack upon Bright's disease, but our efforts will have greater weight and better results if in addition to the care of the individual, the laymen is informed of the nature of nephritis and the care needed to prevent what must above all else be considered a preventable disease.—J. E. GREWIE, *Editorial Lancet-Clinic.*

### THE TREATMENT OF OPHALMIA NEONATORIUM

BY AARON BRAV, M. D., PHILADELPHIA.

Every case of blindness in children resulting from opthalmia neonatorum is a sad commentary that reflects discredit upon the medical profession. Opthalmia in the new-born baby is both a preventable and curable disease, when trated intelligently and early, and no child, especially when delivered by a physician, should be allowed to pay the penalty for the sin of his father with the sacrifice of his eyesight. In these days of medical advancement and preventive science we must not say, "The fathers have eaten sour grapes, and the children's teeth are set on edge." but rather make every effort that "the son shall not bear the iniquity of the father."

Opthalmia neonatorum is still a frequent disease, although not as frequent as it used to be prior to the introduction of the prophylactic measure of Crede; and it is still the great contributor to the causation of blindness. Very often of course we must attribute the trouble to the ignorance of midwives, but not infrequently we also find it as a result of carelessness on the part of the attending physician.

Gonorrheal ophthalmia in the new-born must be considered as a very grave affection. If not treated early and intelligently it always causes total blindness and forever destroys the usefulness of the unfortunate and places him at the mercy and expense of the state.

*Preventive Treatment.*—When we recall the simple fact that it is estimated that in Europe there are about 300,000 cases of blindness, and in America about 35,000, as a result of this disease, the need of using all preventive means becomes self-evident. This can be done effectively without medlesome governmental interference. Somethow, I cannot favor too much governmental authority over the affairs of men, excepting of course in cases in which there is danger to the rest of the community. I believe in the potency of educatino on the subject by a properly constituted body—either the board of health or the society for the conservation of vision—among the lay public would greatly tend to reduce the danger of blindness. Reporting all cases of ophthalmia in the new-born has been and still is advocated in many circles. This is a sound measure, employed properly for the purpose of obtaining information as to the frequency of the disease. This would also, I believe, prevent some laxity that may lurk with the obstetrician or midwife. Any further interference by the authorities, however, is unnecssary and is against the best interests of the profession.

The use of Credé's method as a prophylactic agent is of course a valuable aid in the prevention of this disease. This has been satisfactorily demonstrated and needs no repetition here. The solution need not be stronger than two per cent. I have seen recently a case in which a physician has instilled several drops of a five per cent. solution of silver nitrate with very unpleasant results. Such strong solutions may cause a destruction of the superficial epithelium and thus open an avenue of corneal infection which may ultimately produce blindness. In private practice, no doubt, there is great laxity in the use of this preventive measure; many physicians do not think it necessary to use the Crede method. In fact, they have delivered so many cases without ever meeting one case of ophthalmia that they naturally forget all about its existence until reminded unpleasantly by its occurrence. The majority of midwives do not use it It would be well for more authoritative body to call the attention of midwives every six months, by properly prepared literature, to the necessity of using a two per cent. solution of silver nitrate in every case, no matter whether they suspect gonorrhea or not. In fact, they could be supplied with the solution. They could also be instructed in the danger of this disease—that it is not merely a "cold in baby's eyes," and as soon as they see any discharge or inflammation in the eye, they should insist upon calling in a physician and explain to the parents the necessity of prompt action in the matter. In thus taking the midwife into our confidence I feel a large number of cases could be saved from bindness by early action. I think this would be productive of better results than meddlesome interference on the part of a young mdical inspector, clothed in some garb of authority. Obstetrical cleanliness is of course a factor in reducing the number of cases of opthalmia.

The eradication of that wide-spread social disease gonorrhea would of course be the ideal prophylactic measure. But is this possible? This difficult problem is at present receiving considerable attention from both the medical and the lay public, but no one has as yet been able to discover any possible means for the accomplishment of this desired end. Nor will various workers in this field ever unite. There is a variation in the degree of the sexual thermometer between those who seek the reform, and the eradication of the disease, and those who seek the gratification of the sexual impulse, that lies at the bottom of all the difficulties in dealing with this problem. It is easy, comparatively easy, for the people who have reached the freezing point of their sexual thermometer to discuss calmly matters pertaining to the sexual question (and the psychologist may even suspect some sexual pleasure in the very discussion of the subject), but it is doubtful whether those whose sexual thermometer as a natural consequence of youth and vigor has risen to the boiling point will be guided in their sexual life by the opinion of those whose sexual life has reached the climacteric.

Early marriages would be the most rational method in dealing with the social aspect of the disease. Early marriages (without any medical certificate) would be the best preventive means, and public opinion could probably be moulded in that direction. This is for the future to decide, however, while we are concerned with the present. The best prophylactic measure at present is education of the midwife and the public. The millennium has not yet come. Preventive medicine, while it has accomplished very much indeed in its effort to eradicate disease, has until now not altogether been successful. Traces of the existence of ophthalmia and its serious consequences are still noticed everywhere. It still constitutes the principal element in the causation of blindness. It is true that not every case of ophthalmia in the newborn is of gonorrheal origin. Only in about seventy-five per cent. will the gonococci be found. Scientifically speaking, only the presence of the gonococci in the conjunctival discharge justifies the diagnosis of gonorrheal ophthalmia. From the therapeutic standpoint, however, the attending physician should consider every case of conjuctical inflammation occurring within the first two weeks after birth in the new-born baby of gonorrheal origin, and at once institute such measures as will tend to save the child's eyes. He should not wait for microscopic evidnece to corroborate his suspicion. It is always best to err on the safe side and give the child the benefit of the doubt. Do not ask the father to acknowledge sinful conduct; no man is willing to give evidence that will incriminate himself. His admission is of very little help; his denial absolutely useless. Proceed at once as a clinician on clinical evidence and remember that the sight of the child is at stake. Overtreatment in these cases is apt to bring less dangerous results, in case the condition is non-gonorrheal in origin, than undertreatment would where the disease is caused by Neisser's gonococci. Overvigilance may inconvenience the family to some extent, especially when they cannot afford to have a trained nurse, but it will save many a little one from blindness and a subsequent life of misery. Always give a clear statement on the subject to the parents. Do not undertake the condition. You must explain to the family the nature and danger of the disease and the necessity of proper care.

When called to see a case of ophthalmia in the new-born it is well if possible to determine the exact condition of the cornea so as to be able to give an intelligent prognosis to the parents. One must, however, be very careful, for often harm may be done. The lids must be separated carefully, and should be done only when they yield rapidly to the traction of the physician. Never try to force the lids open when there is marked swelling and plepharospasm. Wait a day or two, if necessary, until you have reduced the swelling, so that you may open the lids without injuring the cornea. Some nurses have a mischievous habit of forcing the lids upon with a hairpin. Always caution her against it. Never use lid retractors to separate the lids; they are dangerous even in the hands of the expert. When on account of swelling of the lids and plepharospasm you find it advisable not to force the lids apart to enable you to inspect the cornea, explain to the parents the condition and tell them it will take a day or two before you can give a definite prognosis. As soon as the swelling has been reduced and the cornea inspected, the people can be informed of the probable outcome of the disease. Parents are very anxious in these cases for a statement as to the prognosis. If the cornea is found intact, and vigorous treatment can be followed out, the people can be informed with almost absolute crtainty that the ultimate result will be good. If there is a corneal involvement the prognosis must be considered grave as far as vision is concerned. This is absolutely necessary where the case is treated by a general practitioner, for in his hands corneal involvement during an attack of gonorrheal ophthalmia is absolutely sure to result in vision being entirely lost. In the hands of the specialist this will depend upon the degree of corneal involvement and surroundings of the case.

In treating a case of ophthalmia one must also remember that it requires about four weeks before the patient may be discharged as cured. Do not stop treatment too early. I have seen cases in which because of a general subsidence of symptoms the physician became lax in his effort after a few days' treatment, only to find an exacerbation with corneal involvement and the subsequent loss of one eye. Every case in the management and technique counts in these cases and should be duly exercised. Cold compresses are an important feature in the treatment of the disease. They are as an antiphlogistic agent, reducing the inflammation and the swelling considerably and relieving the blepharospasm. Very often when the eyelids cannot be separated at the first visit, after cold compresses have been applied for a day or two the spasm of the lids has ceased and the physician is able to inspect the cornea. These cold compresses must be applied constantly day and night for the first three or four days, and they must be changed every five minutes. The discharge is usually lessened after cold compresses have been applied for a time. It is not probable that the reduction in the local temperature as a result of the cold compresses has any deleterious effect upon the gonococci, yet this much is sure, that their antiphlogistic power has a beneficial influence upon the treatment, probably by increasing the local resisting power of the part. Cold compresses, however, have another influence, and that is that they draw the parents' attention to the important of the fact of the gravity of the disease. It also acts as a good reminder for the application of medicinal agents, which might otherwise be omitted, several times during the day, and especially in the evening.

Considering the medicinal agents proper, it is well to call attention to the fact that it is not the kind of agent that you use that will determine the success or failure of your treatment, but how it is used.

Antiseptic lotions are essential, not so much for their germicidal as for their cleansing effect. It follows that no matter what agent is employed it is the frequency and the thoroughness with which you employ it that really do the work. The best cleansing agent in the beginning in my hands I find to be a 1:10,000 bichloride solution to irrigate the conjunctival sac every half-hour for the first two days. I do not rely upon the antiseptic value of the medicinal agent, but upon the frequency of its employment and upon its cleansing property, As a matter of fact, sterile water would accomplish the same result. It is after all the cleansing part we are after, so as to remove every vestige of discharge. I employ the above solution only with the knowledge that I probably have a germ-free solution as my irrigating medium. It is essential in the beginning to irrigate the conjunctival sac every half-hour, for ni these cases it is surprising how quickly the discharge reforms. One must be careful to remove every bit of discharge and use the eye-wash as long as the desired end is obtained. I believe that the best way to accomplish this is with the eye-dropper. I do not employ the fountain syringe; it is not necessary and very often more attention has to be paid to controlling the fountain syringe than to the thoroughness of the irrigation. Espcially in private practice the best and most reliable medium is the eye-dropper. I have seen a case in consultation where the family used the fountain syringe, which was as filthy as it could possibly be, and had been used for all other purposes. The syringe had to be controlled, so that the physician who had to wash the case really only got the solution on the surface, not being able to open the lids, his efforts being concentrated on the control of the syringe. Needless to say the child was hopelessly blind when I saw the case.

The dictum in irrigating the eye must be wash, and wash, and wash again. After all, the frequent washing and removal of the discharge is the only safeguard against corneal infection. It is also advisable to give a 25-per cent. solution of argyrol to the family to instil immediately after washing the eye. The effect of argyrol is probably only due to the fact that, being a heavy substance, it goes all over, diffusing in the conjunctival sac and bringing up infected material to the surface, which of course is again washed away at the subsequent irrigation. It may have some antiseptic properties, but I do not rely upon it as the all-active germicide in the treatment of this disease.

The remedy *par excellence* from the point of view of a germicide is the old standby, silver nitrate. It has stood the test of time and no effort has been able to displace it. Always remember silver nitrate in connection with ophthalmia neonatorum and never for once permit yourself to be misguided by some luring advertisement for a substitution. You do not need a substitute when you can have the real article at a low price. Neither protargol nor argyrol, sophol or collargol, or any other silver preparation, should be relied upon, although they may be employed as adjuncts in the treatment. Silver applications must be made by the physician himself, and should not be trusted to either the family or even th trained nurse. The application should be made once daily. Do not instil the solution into the conjunctival sac, but rather use an applicator—a toothpick wrapped with a piece of absorbant cotton is a good applicator. The strength of the silver solution should be one per cent. The application should be made with care. Evert the conjunctiva gently and just smear the conjunctival surface with the solution. Avoid touching the cornea. The silver soon forms an albuminate layer over the conjunctiva, imprisoning as it were in its meshes the gonococci, which are soon eliminated with the shedding of this albuminous layer in the discharges, and which can be then easily washed away during the process of irrigation. After a few days' treatment the swelling of the conjunctiva is reduced and all the discharge is lessened. I am in the habit of following up this treatment for one week. I then discontinue the bichloride solution and substitute a solution of zinc sulphate one grain to the ounce to irrigate the conjuctival sac every hour or two, as the case may require it. This is done for the reason that complicated with corneal involvement, when of course the treat-very often there is a mixed infection, which readily yields to the zinc solution. I use this for one week, and continue the silver application and argyrol instillation. By this time the eye is usually in a quiet state. I order now a saturated solution of boric acid to irrigate the eyes three or four times daily for about two weeks, when the case is ready to be discharged. Occasionally, however, the condition, as a result of indifference, or neglect, may be ment has to be changed

Once corneal involvement is suspected the case should be turned over to the specialist, for corneal complications in ophthalmia neonatorum in the hands of the general practitioner are sure to result in total blindness. It is probably much easier to prevent corneal complications than to cure them. The experienced man, however, may obtain fairly good results and save useful vision even when there is corneal involvement. As soon as corneal ulceration is suspected the cold applications must be dispensed with and substituted by moist warm compresses. The cold compreseses in such cases have a deleterious influence upon the corneal tissue, reducing its resisting power and thus favoring necrosis of the cornea. On the other hand, the warm moist compress stimulates the corneal tissue, increases its resisting power, and also acts as a nutrient to the corneal. It also reduces pain, which is always present in corneal ulceration. When the cornea is ulcerated it is best to use a simple non-irritating eye lotion. This lotion should be instilled preferably while somewhat warm. As in all other corneal diseases, atropine is an essential drug and should be instilled three times daily. The application of silver nitrate should continue and is not contraindicated. If there is any ulceration, the best thing to do is to use iodoform powder daily dusted upon the ulcerated area. Dionin powder is a useful adjunct, especially when the ulcer is beginning to heal; it greatly reduces the opacity. Cauterization of the ulcer I find not to give the best result.

Operative procedure is contaminated in these cases. In some cases the disease advances until the cornea is destroyed, perforation takes place, and a general opthalmitis sets in. More favorable cases leave the eyeball, but with very large leucomas and vision absolutely *nil.*

Should these cases of ophthalmia be treated at home? Yes, under all circumstances. These children do better at home than in the hospital. There is no need for a special well-trained nurse, who very often is a mere ornament in the house. Any woman with common horse sense, if given proper instruction, can do the work. As a matter of fact hospitals do not admit such cases. If the child is admitted without its mother, the difficulty of feeding is super-added to the disease; if the mother is admitted and has to stay there for a month or longer, the inconveniences of the home must be considered, for there are probably other members at home that need the mother's help. Wherever possible I believe these cases should be treated at home, and they will have a good chance to recover.—*Therapeutic Gazette.*

---

### THE ROLE OF DISINFECTION, AND THE INFLUENCE OF INFECTED ROOMS AND FOMITES IN THE TRANSMISSION OF INFECTIOUS DISEASE.*

By Alvah H. Doty, M. D.

New York

In order that this subject may be scientifically and properly considered, it is necessary that we should be familiar with the means by which infectious diseases are transmitted, for without this knowledge disinfection and other methods of protection cannot be intelligently carried out.

No theory has been more generally accepted, both by the medical profession and the public at large, than that infectious diseases are commonly transmitted by clothing, baggage, money, rags, and innumerable other articles which are supposed to convey pathogenic organisms in their active state from one person to another. These alleged agents of infection are known as fomites. Even a superficial investigation reveals the fact that the origin of this theory antedates the period of bacteriological or other scientific research, for even as early as the fourteenth century we find that clothing and cargoes of vessels were frequently exposed to the air and sun for an indefinite period for so-called purification, and vessels departing from presumably infected ports were sunk by official order to prevent infection through the medium of their contents. These extreme and unjustifiable methods have not by any means been confined to the past, for in recent years, and even at the present time, sanitary regulations are enforced, particularly in regard to disinfection, which are worthless and unnecessary. It was not so very long ago that iron rails were disinfected in the south to prevent the transmission of yellow fever. Since the discoveries of Pasteur and Koch, which gave to the world indisputable evidence of the germ origin of infectious diseases, and particularly during the past fifteen or twenty years practical sanitarians have been slowly but surely accumulating conclusive evidence of the fallacy of the fomites theory, and although exceedingly valuable pioneer work has been done in this direction, the results have been reluctantly accepted, chiefly on account of the popularity of this belief, which was born in ignorance and has had little to support it beyond the fact that it offers a ready and plausible explanation for the transmission of infectious diseases in instances where the true medium of infection is unknown. Than this no theory ever entertained has been so pernicious, or has militated so seriously against the successful treatment of outbreaks of infectious diseases, for it has discouraged the employment of active, energetic, and exhaustive measures to detect the true cause of infection. Those in charge of the public health, while they have been conscious of its shortcomings, have been disinclined to abandon it, for it has dominated all health regulations, the object of which is to prevent infectious diseases. However, in recent years, events have occurred which fortunately have abruptly and positively demonstrated the unsoundness of this theory, and have extended valuable aid in bringing about a more serious consideration of this important subject, for we have learned that the ideas were formerly entertained regarding the means by which the various infectious diseases are transmitted, are wrong, and it seems to me there can be no better time than the present to condemn the fomites theory and accept the results regarding the transmission of infectious diseases which have obtained by careful and painstaking scientific investigation.

Not long ago malaria was attributed to the presence of miasmata or poisonous vapors emanating from swamps; now we know that this disease is caused only by the mosquito. We also know that yellow fever, formerly supposed to be commonly transmitted by fomites, is also contracted only through the medium of this insect. The medical history of the south is rich in statistics which presume to offer conclusive proof that the clothing of those who have been exposed to yellow fever was responsible at various times for outbreaks of this disease.

Satisfactory evidence has been given us that plauge, which was believed to be caused by almost anything in the way of fomites, is conveyed from one person to another by the rat through the medium of the fleas which infest it. Some years ago I visited Russia during an outbreak of this disease, and found many theories regarding the media of infection. Instances were cited where it was transmitted by caravans going from one section of the east to another, through the medium of rugs, clothing, etc. In the light of our present knowledge, these theories are unworthy of consideration.

Many have maintained in the face of the warning which has been given in regard to this belief that typhus fever must surely be caused by fomites and refer to the rapidity of its extension and other details connected with its transmission. It would be difficult to find a text-book which does not in positive terms state that typhus fever is transmitted by clothing, baggage, and other articles, yet careful investigation, particularly that which has recently been carried on in Mexico under the direction of

Doctor Goldberger and Doctor Anderson, seems to furnish satisfactory proof that typhus fever is not transmitted by fomites, but by the body louse.[1] I originally accepted the general belief regarding the medium of infection in this disease, but my experience during two epidemics has made it quite clear to me that fomites, to say the least, do not constitute the ordinary means of infection. During the outbreak of tyhus fever in New York City in 1892-3, of the 700 or more cases which occurred I remember but two which appeared among persons in the better walks of life, almost all being among the lodging or tenement house population. These people ride in public conveyances with those of other classes, and if clothing constituted a medium of infection, there would be no such record as I refer to. On the other hand, those affected are just the ones we should expect to be the hosts of these insects.

Of the specific organisms involved in smallpox, chickenpox, measles, and scarlet fever we know practically nothing, except probably the information given us by Councilman regarding his investigation of smallpox. There is no doubt that the vesicle or postule of smallpox contains infectious matter; of this we have definite proof. It is asserted by some that infection is also present in the vesicle of chickenpox, although we have no evidence of it. In measles and scarlet fever the skin eruption is of a different character, and in neither disease passes beyond the papular stage, and there is nothing to justify the belief that it contains infection. The desquamation which follows the eruption of these diseases has been regarded as an important medium of infection; this also is without satisfactory evidence. Desquamation in itself has no special significance other than to indicate some circulatory disturbance of the skin or some other interference with its function. Filth causes it. It occurs in sunburn and sometimes in typhus fever and other infectious and noninfectious diseases.

I believe that desquamation is a negligible factor in the transmission of measles and scarlet fever and that these diseases are conveyed from one person to another by the infected discharges of the mucous membrane involved. We have so concentrated our minds on the presumed potency of desquamation as a medium of infection that it has obscured the real danger of the infected discharge which are always present in these diseases. I am aware that instances are cited where it would appear that desquamation is the only logical means of infection in secondary cases. Before this can be accepted as scientific or practical evidence, the presence of infected discharges must be excluded. I do not believe this can be done, nor do I believe this is usually considered. I know of many who regard the discharge from the affected membrane in scarlet fever and measles as of secondary importance to desquamation in the transmission of these diseases. Proof is presented that measles and scarlet fever are both transmitted before and after desquamation. besides it is far more in accord with modern scientific research to believe that the seat of infection is in the mucous membrane involved rather than desquamating scales:

We probably do not fully consider to what extent the fomites and desquamation thories depend upon statements made by the laity. There is hardly a family, particularly where there are children, that cannot cite instances to prove, to its own satisfaction at least, the transmission of these diseases through the means just referred to, and it will often present evidence that it has occurred through the medium of clothing which has been laid away for months or even years. Equally positive statements are made regarding the transmission of disease by desquamation. I do not mean that all evidence of this character is presented by laymen. I have investigated much of this evidence and have yet to see the first case where satisfactory proof of these statements was available or a condition which could not be explained without the aid of the fomities or desquamation theory; still, under these uncertain conditions we have gone ahead in the investigation of disinfectants and disinfection; we have indicated in detail how the apartments of the sick must be treated and what agents should be employed; it does not seem proper that we should do this until every means is exhausted to ascertain the true media of infection. We construct a foundation before we build the house, and for the same reason we should so far as possible know the manner in which infectious diseases are transmitted, in order that we may have a proper knowledge of the methods by which we can best prevent them.

It is only within recent years that we have appreciated the importance and danger of mild, ambulant, or irregular cases of infectious diseases and the frequency with which they occur. I regard them as one of the most common and dangerous factors in the transmission of infection, because they often pass unrecognized and are responsible for outbreaks of disease which are attributed usually to fomites. More recently we have learned of "carriers" or persons who, although they present no evidence of disease themselves, bear specific organisms and may transmit them to others. With a proper knowledge of the means of infection before us, it is not necessary to depend upon either the fomites, desquamation, or the aerial theory to aid us in determining the manner by which infectious diseases are conveyed. I mean by the aerial theory, the belief that infection is carried for long distances through the air. It is frequently asserted that this may occur, even to the extent of half a mile or more. I do not believe in this theory or that there is any substatial or reasonable proof of it.

Until within a few years it has been generally believed that the marked increase in the number of cases of scarlet fever, measles, and diphtheria which occur at the opening of the school season is due largely to the transmission of these diseases by the clothing of the children in whose home infectious diseases exist, or who have been exposed to these conditions outside of their homes. This belief has been shattered by the work recently carried on in the schools of the larger cities. Here the careful inspection by medical officers, known as the School Corps, has shown to what a remarkable extent mild, ambulant, irregular, or unrecognized cases exist. Children with a slight sore throat which has not materially interfered with their play or school duty, or the presence of a nasal or ear discharge, has frequently been found to be due to a mild case of scarlet fever or dipththeria or "a cold in the head" has proved to be due to measles. Before the existence of this exceedingly valuable inspection these cases were overlooked, and secondary ones were attributed largely to the presence of the infected clothing. Conclusive evidence of the great frequency of "carriers" has also been presented, and I am sure we shall be surprised to learn to what extent they occur and the number of diseases which are involved.

Money has long been regarded as a medium of infection; so have rags, although of this there is no satisfactory evidence. It is the superficial consideration of this part of the subject that has misled us. If an employee of a bank contracts infectious disease it is very apt to be attributed to infected money. If a man who works in a paper manufactory contracts smallpox, the rags with which he deals are usually held accountable for it unless there is positive evidence of some other means of infection. It is important to ascertain at this point if those who work among rags, or those who are constantly handling money, contract infectious disease oftener than others. If not, the evidence does not support the formites theory, for we must remember that those who are in close contact with these articles are subject to the same general exposure as others, and therefore may contract disease in the ordinary way. During a careful investigation of this part of the subject, which has covered a number of years, I have yet to find an instance which reasonably proves that either money or rags constitute a medium of infection. At the treasury department in Washington, where an enormous amount of old and filthy paper money is being constantly handled and rehandled by many employees, no evidence could be secured that money transmits disease. This same result was also obtained in connection with the employees of banks and other institutions where money is being constantly dealt with. Similar results occurred in connection with the investigation of rags, second-hand clothing, etc., for no proof was disclosed to show that diseases are transmitted in this way. My investigation in connection with rags was carried on both in this country and in Egypt, for the reason that Egyptian rags have been used largely in the United States for the manufacture of paper. They usually consist of the cast-off garments of the natives, which as a rule are worn next to the skin; when unfit for further service, they are sold to the rag man or exchanged for pottery for domestic purposes, and are taken to Alexandria and there picked over by women and children. Although infectious diseases are constantly found throughout Egypt, I was unable to detect any evidence in the carefully prepared statistics of the English sanitary officers in charge that rags constitutte a medium of infection.

We are frequently confronted with statements that money contains a large number of bacteria; this is true; they also exist on our hands and faces, on our linen, car straps, balustrades of stairways, and in innuemerable other places, but they are not the kind of organisms that transmit infectious disease. From a bacteriological standpoint a very interesting series of experiments in connection with this part of the subject was made by Mr. Warren Hilditch, of Sheffield Institute, which demonstrated the fallacy of this theory.

Physicians are frequently concerned regarding the possibility of transmitting disease from one patient to another through the medium of their own clothing. Instinctively they know that this does not happen or at least only in rare instances. Gowns are frequently used in the sick room to protect against this possible danger. I am quite sure my readers will agree with me that it would be exceedingly difficult to arrange a practical system by which a physician could always have a gown at the proper time in dealing with the various forms of infectious disease which he meets in his daily practice.

In making applications or otherwise dealing directly with the infected membrane where sudden expulsion of mucus frequently occurs, it is proper that the physician should be protected by some form of covering. In these instances a sheet may be so deftly used as to answer all the purposes of a gown and should afterward be treated with heat in the same manner as are other articles directly connected with the patient.

No one who is practically familiar with this subject doubts that in some rare instances clothing, money, rags, or other articles may act as a medium of infection; while this should be given proper consideration, we must devote our time, in public sanitary work, rather to the usual or common means of infection.

If our former theories regarding the means by which infectious diseases are transmitted, are wrong, what are the real means of infection?

The knowledge we may possess on this subject proves that infectious diseases are transmitted by persons rather than things; by contact with others; by certain discharges of those who are infected; and by insects and vermin.

We are familiar with the fact that in typhoid fever and cholera it is the intestinal discharge, and in diphtheria the discharge from the nose and throat, that we must deal with in the protection of the public health, and we know that when this condition is properly looked after, the most effective and satisfactory results follow. However, in measles and scarlet fever we have dealt rather indefinitely with the subject of infection, for this bugaboo of desquamation has weakened our lines of defense against the real danger—that is, the infected discharges. As the specific organism of these diseases has not yet been identified, it is obviously difficult or impossible to determine when the discharges cease to act as a medium of inspection; still, the problem will be much sooner solved if we direct our attention to the real danger instead of the theoretical ones.

In hospitals, where measles and scarlet fever as well as other infectious diseases are treated under the same roof, and the wards containing these various diseases are separated only by low or dwarf partitions which leave the upper part of the wards continuous with each other, there is practically no transfer of infection from one ward to another, neither is room disinfection carried out as a part of the routine work. These results certainly do not favor the desquamation or aerial theory of infection, and it would seem that, when careful attention is given to the disinfection or destruction of the discharges from the nose, mouth and ears and above all where general cleanliness is strictly observed, which I believe ranks higher than disinfection, we have done all that modern sanitation calls for in the treatment of this condition.

Measles and scarlet fever, as well as other infectious diseases, are commonly conveyed from one person to another through the medium of infected hands. Dipping the hands for a moment in a disinfecting solution does not disinfect them, for it takes some time to penetrate the infected material. It is the proper preliminary cleansing of the hands with soap and water upon which protection in this direction really depends.

So far as the trasmission of infectious diseases by insects is concerned, it may be said that the investigation of this subject, although yet in its infancy, has already yielded results which have been of great benefit to mankind, and it is fair to assume that there are many other diseases and other insects concerned in this manner of infection, of which as yet we have no knowledge. I believe this calls for every effort on our part to eliminate the mosquito by destroying its breeding place, and that the same action should be taken in regard to the fly or other incests which we suspect as agents of infection. But little will be gained in the way of eliminating them by efforts to destroy the winged insects. It is the destruction of their breeding places that must receive our careful attention.

I have dwelt at some length upon these details, because we cannot properly protect against infectious diseases unless we are familiar with the means by which they are transmitted. With this knowledge it is comparatively easy to deal with almost any condition which may exist; besides, the work of protection is much more simple and more easily carried out.

Probably no sanitary measure has been so variously dealt with as disinfection. The methods employed are frequently unnecessary and not in accordance with the dictates of modern sanitation. Agents are used which have little or no value, material in household or on shipboard is uselessly injured or destroyed, and when disinfection is actually required it is frequently imperfectly carried out. I may take for instance the treatment of the discharges of typhoid fever. The method usually employed consists in placing a disinfectant in the bed pan before or after the discharge, the mixture being allowed to remain for some time before the receptacles is emptied. This method cannot be depended upon for thorough disinfection; besides, not infrequently in handling or manipulating the receptacle the nurse or attendant is infected, for she has faith that this treatment answers all requirements. Some time ago I made a series of experiments in regard to this detail. They consisted in the treatment of fecal matter with the various disinfecting solutions which are commonly recommended for this purpose, and in the manner in which they are usually employed. It was found at the end of twenty hours that but one-eighth of an inch of the mass was disinfected. These experiments were subsequently repeated by others with practically the same results, and I believe they prove that this method of treating the discharge does not disinfect, and it is not improbable that when the fecal matter is thrown in the water closet or privy vault and disintegrated, it may later infect something which is taken into the mouth.

It is true that the discharge of cholera becomes rapidly fluid and also more quickly succumb to disinfecting agents, still these conditions are sometimes uncertain, and we should make no exception when we have means at our command to secure full protection. Furthermore, it is exceedingly important that the receptacle itself should be disinfected at the same time the discharge is treated, although the attendant does not fully appreciate this and sometimes does not realize the necessity of cleaning the hands. I believe there is but one way to deal with infected discharges of this kind if it can be made use of, and that is by heat, either boiling water or steam. Some simple means of performing this may be improvised wherever a metal receptacle of sufficient capacity and a fire can be secured, or a simple and inexpensive apparatus may be made as follows: A sheet copper receptacle, sufficiently large to hold a full size bed pan may be constructed, having metal supports to raise it above the ground high enough to allow room for a lamp or gas apparatus to secure the necessary heat. The cover should, if possible, be made sufficiently heavy to offset a slight pressure of steam. This, however, is still further provided for by a spout which it attached to the portion of the top not involved in the cover, and for the same purpose that a spout is used on a tea kettle, to allow the escape of steam. The upper end of this should be connected with a flexible tube which may be carried out of the window in order that the steam does not escape into the apartment itself. When not used the temperature of the water may be kept short of the boiling point. The addition af a small amount of potassium permanganate will usually prevent any unpleasant odor. The value of this method lies in the fact that when the bad pan is brought from the patient, placed in the bath, and exposed to boiling water for twenty minutes, we may be certain that both the pan and the discharge are disinfected, and it makes little difference what is done with them afterward. The apparatus is only a suggestion of the principle, which may be carried out on a larger scale. In the hospitals which have been under my direction, large apparatus were constructed along these lines, capable of holding eight bed pans at one time. Steam may be used instead of boiling water. This method of disinfection is valuable in any form of infectious disease, for the treatment of discharges or textile fabrics or other articles which may have been directly contaminated.

I am aware that it is possible to disinfect with carbolic acid and other agents, and in some instances it is not practical to use boiling water or steam, but when it can be employed it should take precedence over all other agents. It offers full penetration, its action is certain and prompt, it does not involve danger or expense, and apparatus for its use can almost always be improvised; besides, it is the natural disinfectant and will sooner or later be so considered.

A careful study of the means by which infectious diseases are transmitted will teach us that the necessity for disinfection is confined chiefly to infected discharges and articles about the patient that may be directly contaminated; that room disinfection is an unimportant factor in the prevention of infection, that it is employed largely in connection with the fomites theory, and is supposed to represent a final clearing up at the termination of the case. The truth is, we should deal constantly with the infection which is present in order that there may be no terminal disinfection necessary. It is the dependence that is placed upon this which favors carelessness on the part of the nurse or attendant, for the great importance of continued surveillance is not so fully appreciated.

There are instances where extreme carelessness has occured in the treatment of infectious diseases which may justify the disinfection of the apartment. In smallpox we have proof that the vesicles contain infectious matter, and it is the general practice to disinfect the apartment after the patient is removed, although there has been strong evidence presented to show that after the removal of the patient, the room ceases to be a medium of infection. I am willing to say that I am not unfavorable to this opinion, and I believe that the statements which have been handed down to us, describing the infection which is constantly lurking about the apartment where infectious disease exists in the form of organisms in their active state, is largely a creature of the imagination. We believe this largely by inference. I have no desire or intention to overlook any proper or reasonable means of protection, but I believe room disinfection goes hand in hand with the fomites theory, and that both in various ways encourage carelessness; they are discredited and should be replaced by modern methods which, I feel certain, offer far greater value.

It may be said that the gases which are used for room disinfection—sulphur dioxide and formaldehyde—have very little power of penetration and can be depended upon only for the most superficial form of disinfection. Furthermore, the sprinkling or spraying of rugs or carpets which may have been contaminated with discharges, is not a safe or practical method of disinfection. If these articles are contaminated they should be subjected to heat, either boiling water or steam.

I believe that nothing is more unreasonable than an attempt to disinfect an entire house, and I can imagine no condition that would call for it, unless it is to cater to the wishes of the family.

It is impossible in the limited space given this paper satisfactorily to consider the many details connected with this important subject. I believe the essential ones are that we now have conclusive proof that many of our former theories regarding the means by which infectious diseases are transmitted are wrong, and that in the prevention of these diseases we should in the future carry out such means as are dictated by our present knowledge of this subject, and that theories and measures which are not supported by scientific evidence and practical experience should be discarded.

If this course is followed, we may as a rule expect to detect the origin of outbreaks of infectious disease, which constitutes the most important and valuable means of dealing successfully with this danger. Further means of protection include the prompt and careful isolation of the patient and the constant disinfection or destruction of infected discharges and articles which may be directly contaminated. The most careful attention must be given to cleanliness. Than this nothing more important, not only for its great value in the prevention of infection, but for its educational value also.

When the character of the disease calls for it, exhaustive effort should be made to destroy infected insects and to extend general aid in destroying their breeding places.

The treatment of those who have been exposed to infection consists in at least a daily examination early to detect the invasion of the disease. This is not complete without the use of the thermometer. The practice of quarantining well persons in whose homes infectious disease exists should disappear with the fomites theory. The danger of these people is that they may contract disease and transmit it by personal contact, and not that they may transmit it to others by their clothing; besides, it is best that they should be out of the house; furthermore, these people are often the bread winners of the family, and their enforced idleness is a matter of considerable concern. —*New York Medical Journal.*

